# Influence of Sweet Sorghum Silage and Slow-Release Urea on Lamb Meat Quality and Fatty Acid Profiles

**DOI:** 10.3390/foods15091463

**Published:** 2026-04-22

**Authors:** Mingxing Shao, Ziheng Zhang, Rui Li, Liya Zhu, Lanlan Ding, Qing Zhang, Bo Wang

**Affiliations:** 1 College of Grassland Science, Qingdao Agricultural University, Qingdao 266109, China; 15288805403@163.com (M.S.); 19863739296@163.com (Z.Z.); 15209469113@163.com (R.L.); 15761418373@163.com (L.Z.); lldingqq@qau.edu.cn (L.D.); zqing1988@126.com (Q.Z.); 2 Shandong Key Laboratory for Germplasm Innovation of Saline-Alkaline Tolerant Grasses and Trees, Qingdao 266109, China

**Keywords:** lamb, sweet sorghum silage, slow-release urea, meat quality, fatty acids

## Abstract

This study investigated the interactive effects of silage type (corn silage, CS vs. sweet sorghum silage, SS) and nitrogen source (soybean meal, SM vs. slow-release urea, SRU) on lamb meat quality. Results indicated that silage type minimally affected basic chemical composition, although CS-fed lambs exhibited higher ether extract content. Compared to CS, the SS group displayed higher redness (a*) and enhanced antioxidant capacity. SRU improved meat tenderness by reducing Warner-Bratzler Shear Force (WBSF) by 20.40%, with limited effects on other quality traits. Notably, fatty acid profiles and health indices (IA, IT, HH, and HPI) were significantly modulated by the silage × nitrogen interaction. Specifically, the SS diet increased polyunsaturated fatty acids (e.g., LA, EPA, DHA) and achieved a 25.44% higher fish lipid quality (FLQ) value. Crucially, while SRU substitution in the SS diet showed no adverse effects on health value, it detrimentally affected these indices in the CS diet. In conclusion, sweet sorghum silage enhances meat quality and offers superior health benefits, and while SRU improves tenderness, its application requires caution in CS-based diets due to potential negative impacts on nutritional value.

## 1. Introduction

Lamb meat is characterized by its high content of amino acids, vitamins, fatty acids, and minerals, making it a valuable product for human nutritional health [1]. As the world’s largest consumer of sheep meat, China saw its production rise from 1.57 million tons (22.14% of global production) to 2.8 million tons (24.37% of global production) during this period (data from FAO: https://www.fao.org/faostat/en/#data/QCL/visualize accessed on 26 December 2025). Investigation results revealed that about 86% of the Chinese population prefer lamb meat, and consumers were primarily concerned with quality, paying more attention to it than to price [2]. Therefore, meat quality has become a key factor influencing lamb consumption. As reported by Prache et al. (2022), meat quality evaluation encompasses six dimensions: organoleptic, nutritional, safety, commercial, technological, and image [3]. Quality is influenced by factors such as animal characteristics (e.g., breed, age, and sex), on-farm and preslaughter management (e.g., diet, castration, and stress), carcass processing (e.g., chilling and electrical stimulation), and storage (e.g., ageing and packaging) [3]. Among these factors, diet affects all meat quality attributes, particularly nutritional and technological quality [1,3,4]. Therefore, improving lamb meat quality through dietary factors is an important approach to meeting consumer demand for high-quality meat.

Silage is a major roughage component in ruminant diets. Feeding different silages influences not only production performance but also product quality. Sweet sorghum is characterized by high stress resistance, high biomass yield, and good feeding quality, making it a promising silage feedstock to substitute for corn silage in ruminant production [5,6]. Nutritionally, sweet sorghum silage (SS) typically contains higher water-soluble carbohydrates but lower starch and apparent nutrient digestibility than corn silage (CS). However, studies in sheep have reported no differences in feed intake, total-tract nutrient digestibility, or rumen fermentation compared to corn silage [7]. Conversely, other research indicates that replacing CS with SS can lead to lower feed intake and average daily gain—though without affecting carcass traits—while offering better cost efficiency [8]. Similarly, lambs fed with SS exhibited lower growth performance and less carcasses fat, yet possessed better meat nutritional quality [9]. Notably, however, a partial replacement (50%) of CS with SS in goats has been shown to improve growth performance and meat chemical composition (such as protein and fat contents), which also enhances tenderness and nutritional value [10]. Therefore, although sweet sorghum silage is considered a potential substitute for corn silage, its effects on small ruminant production—particularly meat quality—remain not well understood.

Nitrogen is another crucial dietary nutrient. Ruminants meet their nitrogen requirements through plant protein and non-protein nitrogen (NPN) sources, thereby supporting growth regulation and metabolism processes. It is well established that NPN can be appropriately added to ruminants’ diets to replace plant protein (such as soybean meal, SM). Conventional NPN sources, such as urea, are rapidly degraded by urease, leading to excessive ammonia production that can exceed microbial utilization limits and pose a risk of ammonia toxicity. Conversely, slow-release urea (SRU) employs physical coating or chemical modification to retard nitrogen release [11]. This approach not only enhances nitrogen utilization efficiency but also mitigates the risk of ammonia poisoning, positioning it as a safe and effective NPN source. Extensive research on the application of SRU in ruminant feeding has primarily focused on growth performance, nitrogen metabolism, rumen fermentation, and microbial flora. Dietary supplementation with 1% SRU has been shown to increase feed intake and nutrient digestibility, promote the synthesis of rumen microbial protein, and not adversely affect the health of sheep [12]. When lambs are fed low-quality roughage, SRU can provide a sustainable nitrogen source for microorganisms, thereby improving fiber digestibility without compromising growth performance [13]. Similarly, studies in dairy [14] and Angus [15] heifers and dairy cows [16] have demonstrated that SRU supplementation improves nutrient digestibility and influences rumen nitrogen metabolism, while having no negative effects on growth performance. Collectively, these studies indicate that moderate supplementation of SRU as a replacement for plant protein enhances ruminant production without causing significant adverse effects. However, investigations regarding the effects of non-protein nitrogen on meat quality (such as chemical composition, oxidative stability and fatty acid profiles) are limited.

Therefore, the present study aims to investigate the interactive effects of silage type (CS vs. SS) and nitrogen source (SRU vs. SM) on lamb meat quality by evaluating chemical composition, antioxidant enzyme activities, and fatty acid profiles, to provide a theoretical basis for producing high-quality lamb meat using alternative feed sources.

## 2. Materials and Methods

### 2.1. Experimental Design and Treatments

A 2 × 2 factorial experimental design was used to randomly allocate lambs to one of four dietary treatments based on silage type (corn silage, CS; sweet sorghum silage, SS) and nitrogen source (soybean meal, SM; slow-release urea, SRU). The four treatments were as follows: (1) CS + SM, (2) CS + SRU, (3) SS + SM, and (4) SS + SRU. SRU was included at 1% of the total mixed ration (TMR) on a dry matter (DM) basis, replacing soybean meal to ensure that all diets were isonitrogenous. The SRU was sourced from Beijing Yahe Nutrition Co., Ltd. (Beijing, China), with a guaranteed urea content of ≥85% and a nitrogen content of no less than 38%.

### 2.2. Animals and Dietary Formulation

Forty-eight healthy male Hu lambs, with an average age of 3 to 4 months and an initial body weight of 24.11 ± 2.77 kg, were randomly allocated to four treatment groups, with 12 lambs per group. Each experimental lamb was housed in individual pens (2.5 m × 1.5 m × 1 m) and fed individually. The experimental diets were formulated to meet the nutrient requirements of small ruminants [17], and its composition and nutritional levels are presented in Table 1. The basal diet was a TMR with a forage-to-concentrate ratio of 50:50 (on a DM basis). The forage portion consisted of silage and peanut vine at a ratio of 30:20, supplemented with a concentrate mixture. Throughout the 67-day experimental period (including a 7-day adaptation period and a 60-day feeding period), lambs were fed their respective TMRs twice daily at 08:30 and 17:30 on a research farm located at Xinyang Animal Husbandry Co., Ltd., Linyi, China. Feed and water were provided ad libitum. The daily feed allowance (on a DM basis) was adjusted to ensure orts were less than 5% of the feed offered.

### 2.3. Sample Collection

Following the feeding trial, a stratified random sampling method was employed: lambs in each treatment were ranked by weight, paired by similarity, and one was randomly selected from each pair to obtain 6 slaughter lambs close to the group mean. After a 12 h fast, lambs were humanely euthanized by electrical stunning followed by exsanguination. All procedures complied with commercial standards (DB11/T 399-2006) [18]. The pre-slaughter live weights for the CS + SM, CS + SRU, SS + SM, and SS + SRU groups were 37.17 ± 2.89, 38.40 ± 3.15, 34.70 ± 2.11, and 35.88 ± 2.91 kg, respectively (P_silage type_ = 0.061; P_nitrogen source_ = 0.301; and P_interaction_ = 0.983, with corresponding cold carcass weights of 17.71 ± 1.60, 17.82 ± 1.38, 16.23 ± 1.46, and 16.70 ± 1.68 kg (P_silage type_ = 0.072; P_nitrogen source_ = 0.571; and P_interaction_ = 0.624). Immediately following slaughter, muscle samples for enzyme assays were excised, snap-frozen in liquid nitrogen within 30 min post-mortem, and stored at −80 °C until analysis. The carcasses were then chilled at 4 °C for 24 h. Subsequently, the *Longissimus thoracis* (LT) muscle samples between the 6th and 13th ribs were excised from the left side of each carcass. After removing the epimysium and visible fat, the samples were stored at −80 °C for chemical composition analysis. Additionally, a sample of the LT muscle was collected from the same location on the right side; a portion of this sample was used to determine drip loss, cooking loss, and shear force, while the remaining portion was stored at −80 °C for fatty acid analysis.

### 2.4. Meat Chemical Composition

Moisture content was determined by freeze-drying approximately 30 g of sliced LT samples for 72 h until a constant weight was reached [19]. The freeze-dried samples were subsequently used for the analysis of muscle chemical composition. Crude protein content was derived from nitrogen content, which was assayed according to the macro-Kjeldahl procedure using a conversion factor of 6.25 [20]. For ether extract, samples were analyzed using a Fully Automated Fat Analyzer (ANKOM XT15i; Ankom Technology, Macedon, NY, USA) with petroleum ether reflux at 90 °C. Ash content was determined by charring the samples followed by incineration in a muffle furnace at 550 °C until constant weight was achieved.

### 2.5. Meat Quality Measurement

The pH of LT muscle was measured at 45 min and 24 h post-mortem. Measurements were taken by inserting the probe of a calibrated digital pH meter (pH Testr20, Eutech, Vernon Hills, IL, USA) into 2.5 cm deep of muscle cross-section between the 12th and 13th ribs. Color parameters, including CIE L* (lightness), a* (redness), and b* (yellowness), of the meat samples were evaluated with a portable colorimeter (model SC-10, TenovoFood, Beijing, China) after 24 h of chilling at 4 °C. Each sample was measured in triplicate, and the mean value was recorded as the final result. Drip loss was determined according to the method of Honikel (1998) [21]. Approximately 50 g (W1) of the LT muscle was collected from each lamb using a coring device. The sample was packaged in an inflated and transparent plastic bag, ensuring no contact between the sample and the bag. The packaged sample was then suspended in a refrigerator at 4 °C. After 24 h, the sample was removed, gently blotted with absorbent paper to remove surface moisture, and weighed (W_2_). Drip loss (%) was calculated as (W_1_ − W_2_)/W_1_ × 100. Cooking loss was determined by weighing LT samples before and after cooking. Samples were sealed in airtight bags, cooked in a 75 °C water bath until the internal temperature reached 75 °C, held for 15 min, and subsequently cooled under running water for 1 h. Surface moisture was removed from the samples before the final weighing. Warner–Bratzler shear force (WBSF) was measured perpendicular to the muscle fiber orientation using a TMS-PRO Food Texture Analyzer (American Food Technology Combined Company, Glendale, CA, USA) (test speed: 60 mm/min; distance: 40 mm; and trigger force: 250 N).

### 2.6. Enzymes Activity

The activities of muscle antioxidant enzymes, including total antioxidant capacity (T-AOC) and superoxide dismutase (SOD), glutathione peroxidase (GSH-Px), and catalase (CAT) were determined using commercial kits (Nanjing Jiancheng Bioengineering Institute, Nanjing, China) according to previously described methods [22]. The unit definitions for all enzyme activity assays were as follows: for T-AOC, one unit was defined as a 0.01 increase in optical density per minute per gram of protein at 37 °C. A unit of SOD activity was defined as the amount that inhibits 50% of superoxide radical dismutation, with results expressed as units per mg of protein. GSH-Px activity was defined based on the enzymatic reaction rate, specifically as the consumption of 1 µmol/L GSH per minute per gram of protein. Finally, one unit of CAT activity was defined as the decomposition of 1 mmol H_2_O_2_ per minute.

### 2.7. Fatty Acid Profile

Muscle samples were placed into 2 mL EP tubes and extracted with 500 μL of extracting solution (V_Isopropanol_:V_n-Hexane_ = 2:3, containing 0.2 mg/L internal standard, methyl undecanoate (C11:0), Sigma-Aldrich, St. Louis, MO, USA, ≥99% purity). The mixture was vortexed for 30 s, homogenized in a ball mill for 4 min at 40 Hz, and then sonicated for 5 min in an ice-water bath. The samples were centrifuged at 12,000 rpm and 4 °C for 15 min, and the resulting supernatant was transferred to a fresh 2 mL EP tube. The extraction was repeated by adding another 500 μL of the extracting solution to the remaining pellet, vortexing for 30 s, and repeating the homogenization and centrifugation steps. The supernatants from both extractions were combined and vortexed for 10 s. An 800 μL aliquot of the combined supernatant was transferred to a new tube and evaporated to dryness under a gentle stream of nitrogen. The residue was reconstituted in 500 μL of a methanol:trimethylsilyl diazomethane solution (1:2) and incubated at room temperature for 30 min. The solvent was again evaporated under nitrogen. The final sample was reconstituted in 160 μL of n-hexane, vortexed, and centrifuged at 12,000 rpm for 1 min. The clear supernatant was transferred to a Gas Chromatography-Mass Spectrometer (GC-MS) vial for analysis.

GC-MS analysis was performed on an Agilent 7890B gas chromatograph (Santa Clara, CA, USA) coupled to a 5977B mass spectrometer, equipped with a DB-FastFAME capillary column (90 m × 250 μm × 0.25 μm). Fatty acid methyl esters (FAMEs) were identified using a Supelco 37 component FAME Mix (Sigma-Aldrich, St. Louis, MO, USA). A 1 μL aliquot of the sample was injected in split mode (5:1) using helium as the carrier gas at a constant flow pressure of 46 psi. The inlet purge flow was set to 3 mL min^−1^. The oven temperature program was as follows: initial temperature of 50 °C held for 1 min; ramped to 200 °C at 50 °C min^−1^ and held for 15 min; then increased to 210 °C at 2 °C min^−1^ and held for 1 min; finally, raised to 230 °C at 10 °C min^−1^ and held for 15 min. The temperatures of the injection port, transfer line, quadrupole, and ion source were maintained at 240 °C, 240 °C, 230 °C, and 150 °C, respectively. The mass spectrometer was operated in electron impact (EI) mode at −70 eV. Data were acquired in Scan/SIM mode over an *m*/*z* range of 33–400 following a 7 min solvent delay. The limits of detection (LOD) and quantification (LOQ) for the method were determined to be 0.001 g/100 g and 0.003 g/100 g, respectively.

### 2.8. Health-Related Nutritional Parameters

Based on the data related to fatty acid profiles, the health-related nutritional parameters were calculated using the following specific methods [23,24,25,26,27,28]:

Nutrition value index (NVI) = (C18:0 + C18:1n9)/(C16:0)

Index of atherogenicity (IA) = [C12:0 + (4 × C14:0) + C16:0]/(∑ UFA)

Index of thrombogenicity (IT) = (C14:0 + C16:0 + C18:0)/[(0.5 × ∑ MUFA) + (0.5 × ∑n − 6 PUFA) + (3 × ∑n − 3 PUFA) + (∑n − 3 PUFA/∑n − 6 PUFA)]

Hypocholesterolemic/hypercholesterolemic ratio (HH) = (C18:1 + ∑ PUFA)/(C12:0 + C14:0 + C16:0)

Health-promoting index (HPI) = (∑ UFA)/[C12:0 + (4 × C14:0) + C16:0]

Fish lipid quality (FLQ) = 100 × (C20:5n-3 + C22:6n-3)/(total FA)

Note: The abbreviations used in the above equations are defined as follows: UFA, unsaturated fatty acids; MUFA, monounsaturated fatty acids; PUFA, polyunsaturated fatty acids.

### 2.9. Statistical Analysis

Data for muscle chemical composition, meat quality, enzyme activity, fatty acid composition and health-related nutritional parameters were analyzed using SPSS version 22.0 (SPSS Inc., Chicago, IL, USA). The fixed effects of silage type (CS and SS) and nitrogen source (SM and SRU), and their interaction effects were evaluated using a generalized linear model. If a significant interaction was found, simple effects analyses were performed. The model structure is as follows:

Y_ijk_ = u + T_i_ + S_j_ + (T × S)_ij_ + e_ijk_

where Y_ijk_ represents the observed value of the dependent variable; u represents the value of overall mean; T_i_ represents the fixed effect of silage type (i = 1: CS; i = 2: SS); S_j_ represents the fixed effect of nitrogen source (j = 1: SM; j = 2: SRU); (T × S)_ij_ represents the interaction effect between the silage type and nitrogen source; and e_ijk_ represents random residual error. When a significant interaction was detected, simple effects analyses were conducted within each level of the other factor. Results are presented as means and standard error of means, and differences were considered statistically significant at *p* < 0.05.

## 3. Results

### 3.1. Chemical Composition of LT Muscle

The chemical composition of the LT muscle is presented in Table 2. No significant interaction was observed between silage type and nitrogen source on meat chemical composition (*p* > 0.05). The contents of moisture, crude protein, and ash were not affected by dietary silage type or nitrogen source (*p* > 0.05). Lambs fed CS had a higher ether extract content than those fed SS (*p* < 0.05). The partial substitution of SM with SRU showed a tendency to increase ether extract (0.05 < *p* < 0.1).

### 3.2. Meat Quality Affected by Dietary Silage Type and Nitrogen Source

As shown in Table 3, there were no significant interaction effects between silage type and nitrogen source for any measured meat quality parameters (*p* > 0.05). Dietary silage type and nitrogen source did not affect pH_45min_, pH_24h_, drip loss, or cooking loss (*p* > 0.05). For meat color, the a* value was greater in the SS group than in the CS group (*p* < 0.05), whereas L* and b* values were unaffected by either dietary factor (*p* > 0.05). Meat from the CS group was tougher, exhibiting a higher WBSF than meat from the SS group (*p* < 0.05). Conversely, the SRU diet resulted in more tender meat, with a lower WBSF compared to the SM diet (*p* < 0.05).

### 3.3. Antioxidant Enzymes Activity of Meat

The activities of antioxidant enzymes used to evaluate meat antioxidant capacity are presented in Table 4. A significant interaction between silage type and nitrogen source was observed for GSH-Px (*p* < 0.05). Specifically, at the CS level, replacing SM with SRU significantly increased GSH-Px (*p* < 0.05). However, at the SS level, there was no difference in GSH-Px between SM and SRU (*p* > 0.05). Under the SM level, the substitution of CS with SS significantly elevated GSH-Px (*p* < 0.05). Conversely, at the SRU level, GSH-Px did not differ significantly between the CS and SS treatments (*p* > 0.05). The activities of T-AOC, SOD, and CAT in lamb meat were higher for the SS diet than for the CS diet (*p* < 0.05), but were unaffected by nitrogen source (*p* > 0.05).

### 3.4. Response of Fatty Acid Profile to Dietary Silage Type and Nitrogen Source

The fatty acid composition of meat, as affected by dietary treatments, is detailed in Table 5. A total of 32 fatty acids were detected in the sweet sorghum silage group, compared to 30 in the CS group (C13:0, C23:0, and C14:1 n-5 cis were absent in CS group). The concentration of C16:0, C16:1 n-7 cis, C18:1 n-9 trans, C18:1 n-7 cis, C22:4 n-6 cis, SFA, UFA, and PUFA, as well as the SFA/UFA ratio were all significantly affected by the interaction between silage type and nitrogen source (*p* < 0.05). At the CS level, replacing SM with SRU increased C16:0, SFA, and the SFA/UFA ratio, but decreased PUFA and UFA (*p* < 0.05). However, at the SS level, no significant differences in these parameters were observed between the SM and SRU treatments (*p* > 0.05). At the SS level, replacing SM with SRU increased C16:1 n-7 cis, C18:1 n-9 trans, and C22:4 n-6 cis (*p* < 0.05). In contrast, at the CS level, no significant differences in these fatty acids were observed between the SM and SRU treatments (*p* > 0.05). At the SM level, replacing CS with SS significantly decreased C16:0, but increased C22:4 n-6 cis and PUFA (*p* < 0.05). In contrast, at the SRU level, this substitution significantly decreased C16:0, SFA, and the SFA/UFA ratio, while significantly increasing a broader panel of fatty acids, including C16:1 n-7 cis, C18:1 n-9 trans, C18:1 n-7 cis, C22:4 n-6 cis, PUFA, and UFA (*p* < 0.05).

The concentrations of several SFA, including C8:0, C10:0, C12:0, C15:0, C18:0, C22:0, and C24:0, were not affected by silage type, nitrogen source, or their interaction (*p* > 0.05). However, meat from the SS group had higher concentrations of C14:0 and C17:0 but lower C20:0 than the CS group (*p* < 0.05). For MUFAs, the CS group had a higher concentration of C18:1 n-9 cis (oleic acid) (*p* < 0.05). Regarding PUFAs, the concentrations of C18:2 n-6 cis (Linoleic acid, LA), C20:5 n-3 cis (all-cis-5,8,11,14,17-Eicosapentaenoic acid, EPA) and C22:6 n-3 cis (all-cis-4,7,10,13,16,19-Docosahexaenoic acid, DHA) were higher in the SS group than in the CS group (*p* < 0.05). The SS group had lower concentrations of total MUFA, but a higher n-6/n-3 ratio compared to the CS group (*p* < 0.05).

### 3.5. Health-Related Nutritional Indexes

Result analysis indicated that the values of IA, IT, HH, and HPI were significantly affected by the interaction between silage type and nitrogen source (*p* < 0.05) (Table 6). At the CS level, substituting SM with SRU significantly improved IA and IT but reduced HH and HPI (*p* < 0.05). In contrast, there were no significant differences between SM and SRU at the SS level (*p* > 0.05). Under the SM level, the substitution of CS with SS significantly reduced IT and increased HH (*p* < 0.05). Meanwhile, under the SRU level, replacing CS with SS significantly reduced IA and IT, while significantly elevating HH and HPI (*p* < 0.05). The NVI of meat was higher in SS-fed lambs than in CS-fed lambs (0.05 < *p* < 0.1). While the CS group showed a significantly lower FLQ value than the SS group (*p* < 0.05), nitrogen source had no effect on FLQ (*p* > 0.05).

## 4. Discussion

Chemical composition parameters, such as protein, fat, and ash, are crucial indicators for evaluating the nutritional value of meat and are closely associated with its health benefits [29]. Bezerra et al. (2016) reported that the crude ash, moisture, and crude protein contents in high-quality mutton range from 0.79% to 1.68%, 70.80% to 80.25%, and 18.50% to 23.39%, respectively [30]. The nutritional composition observed in all treatment groups of this study was within these reported ranges. More specifically, substituting CS with sweet sorghum silage in the diet reduced the fat content of lamb meat. This may be attributed to the higher metabolizable energy of the CS diet compared to the SS diet, as higher energy levels promote intramuscular fat deposition [31]. Similarly, other studies have found that as the proportion of sweet sorghum in the diet decreases, the dietary metabolizable energy level gradually increases, leading to a linear increase in the fat content of lamb meat [32]. Additionally, the results showed a trend where SRU supplementation increased intramuscular fat content. This increase could be explained by the fact that dietary non-protein nitrogen improved nutrient utilization efficiency, thereby promoting nutrient deposition within the meat in lamb [33,34]. Additionally, while NPN substitution for soybean meal decreases dietary metabolizable energy, it could promote the channeling of non-essential amino acids into lipogenesis, consequently influencing the fat content of livestock products [35]. Additionally, given that the diets differed not only in energy levels but also in crude protein and fiber fractions (NDF and ADF), the potential synergistic effects of these nutritional factors might also have influenced the meat’s chemical composition. However, the specific mechanisms remain to be fully elucidated.

Post-mortem muscle pH is a critical index for evaluating meat quality [36]. The findings indicate that pH decline was unaffected by silage type or the partial substitution of soybean meal with SRU, implying a similar rate of muscle glycolysis among the lambs. Meat color is a key quality attribute of fresh meat, which directly determines consumer acceptance of meat products [37]. The inclusion of SS in place of CS significantly increased the redness (greater than 14.5), and the lightness value also exceeded 44, resulting in a consumer acceptance rate for this meat color of approximately 95% [38]. As sorghum contains abundant polyphenols with potent free radical scavenging abilities, it may enhance the meat’s antioxidant capacity after slaughter, thereby contributing to the higher initial redness observed in this study [39,40]. However, further studies investigating color stability during storage and the specific relationship between color changes and antioxidant substances are warranted to confirm these findings. The sensory traits of meat are closely associated with shear force and intramuscular fat content. Typically, sensory evaluation declines as shear force rises but improves with higher fat content [41]. In the present study, however, lambs fed SS exhibited lower shear force values despite having a relatively lower intramuscular fat content compared to the CS group. This discrepancy suggests that factors determining shear force extend beyond fat deposition. We hypothesize that the difference in shear force was primarily mediated by dietary energy supply. The CS group likely obtained higher energy levels, as evidenced by their significantly higher carcass weights. This elevated energy intake typically promotes muscle hypertrophy and faster growth rates, which are often associated with increased muscle fiber diameter and cross-sectional area. Although muscle structural parameters were not directly measured, this morphological change offers a plausible explanation for the higher shear force observed in the CS group, overriding the potential tenderizing effect of its higher fat content [42].

While non-protein nitrogen, as a synthetic product, possesses significant potential for livestock production, its influence on meat quality remains a critical consumer concern. The study found that supplementing the diet with SRU did not significantly affect pH, meat color, drip loss, or cooking loss. Notably, it decreased the shear force of the meat, contributing to improved sensory attributes. This improvement may be attributed to the ability of SRU to enhance fiber digestibility and overall nutrient utilization efficiency, potentially facilitating intramuscular fat deposition. This proposed mechanism differs from that of silage type; while the specific pathways remain to be fully elucidated, our findings suggest that different dietary components modulate meat tenderness through distinct metabolic routes, warranting further investigation into the structural and biochemical determinants involved. Moreover, the redness and lightness values were greater than 9.5 and 34, respectively, satisfying consumer acceptability thresholds [38]. In a grazing system, dietary supplementation with an appropriate amount of urea did not significantly affect meat color or shear force values at 24 h post-mortem in fattening sheep [43]. Similar findings were also reported in studies where urea or SRU was added to house feeding beef heifers [44] and steers [45] diets, as well as urea in goat [46]. Additionally, other studies indicate that dietary NPN improves cooking loss, shear force, and meat color [47]. Variations in research findings may be attributed to differences in diet type, animal breed, and feeding system [48]. Overall, reasonable dietary supplementation of NPN does not negatively affect meat quality.

Post-mortem antioxidant enzyme activity is critical for maintaining the muscle’s antioxidant defense system and serves as an important indicator influencing the shelf life and stability of meat products. Unsaturated fatty acids, particularly PUFAs, are susceptible to lipid oxidation, leading to product deterioration and posing potential risks to consumer health [49]. Enhancing the antioxidant capacity of meat products helps decrease lipid oxidation of post-mortem meat products. Conventional processing methods employed by meat enterprises, such as drying, fermentation, smoking, and curing, alongside cold-chain logistics, can effectively prolong stability of meat products. Although synthetic additives like butylated hydroxytoluene and vitamin E are commonly incorporated into meat products [50], consumers maintain a strong preference for natural ingredients to increase antioxidant capacity, or for the production of meat with higher antioxidant properties achieved through dietary modulation [51]. Our results revealed that the post-mortem activities of T-AOC and SOD were significantly elevated in the meat of lambs consuming SS compared to those fed CS. T-AOC is an indicator of an organism or tissue’s resistance to oxidative reactions. SOD is responsible for scavenging free radicals, and GSH-Px catalyzes the decomposition of hydrogen peroxide; both enzymes act as a vital defense system against oxidative reactions [52]. The elevated activity of antioxidant enzymes indicates that the meat from lambs fed sweet sorghum silage possesses a more favorable endogenous antioxidant enzyme profile. This suggests that sweet sorghum silage could serve as an effective dietary strategy for utilizing plant-based materials to produce meat with enhanced antioxidant potential. Studies have also reported that replacing corn with sorghum can increase total antioxidant capacity in lambs [53]. This may be attributed to the presence of tannins in sorghum. Although dietary tannins are not absorbed by ruminants, they exert local antioxidant effects within the gastrointestinal tract, thereby favoring the deposition of other antioxidant nutrients and improving the oxidative stability of tissues [54,55]. Furthermore, while substituting a portion of soybean meal with SRU in the CS diet elevated GSH-Px levels in lamb meat, the specific mechanism is not yet fully understood, potentially due to the complex interactions between dietary components.

Fatty acid profile is closely associated with the health attributes of meat products and is a key concern for consumers, especially with respect to unsaturated fatty acid content as well as the composition and ratio of n-6 and n-3 fatty acids [56]. Increasing the consumption of PUFA is beneficial for reducing the risk of heart and cardiovascular diseases [57], and a nutritional recommendation is to maintain an n-6/n-3 ratio below 4.0 [58]. Fatty acid composition varies among different breeds [59], and muscle site and fat content also influence this composition; however, dietary composition remains the primary determinant [60]. Consequently, dietary modulation of muscle fatty acid composition represents the most effective strategy for producing healthier meat products. The results demonstrate that replacing CS with SS decreased MUFA. Furthermore, regardless of the nitrogen source, SS substitution consistently enhanced the PUFA content in lamb meat. Nevertheless, this substitution also led to an elevated n-6/n-3 ratio, indicating that despite the increase in total polyunsaturated fatty acids, n-6 fatty acids increased to a greater relative extent than n-3 fatty acids. Similar studies have also indicated that replacing CS entirely with SS can increase the content of PUFA and decrease MUFA; however, the impact on SFA appears to be breed-related [9]. The ability of SS to increase muscle PUFA levels may be related to its high polyphenol content. Polyphenols like tannins inhibit rumen bacterial proliferation and activity, which attenuates biohydrogenation [61]. Consequently, this increases the amount of UFA escaping the ruminal biohydrogenation and promotes their deposition in muscle. Increasing tannins in the diet at moderate levels can improve the content of EPA, DHA, DPA, total PUFA, and total n-3 and n-6 PUFAs in lamb meat [62]. Apart from the bioactive compounds found in sweet sorghum, variations in dietary energy levels and ingredients are also potential factors contributing to the differences in fatty acid profile. Three other fatty acids (C13:0, C23:0, and C14:1 n-5 cis) were found solely in the muscle of animals consuming SS. This further suggests that silage type modulates fatty acid composition, thereby potentially inducing differences in meat flavor and stability [63].

It is particularly intriguing that despite the lack of a significant main effect of SRU substitution for soybean meal on the overall fatty acid profile, the total SFA, UFA, and PUFA concentrations were significantly modulated by the interaction between silage type and nitrogen source. These finding highlights that the efficacy of SRU in modulating fatty acid composition is not absolute but is contingent upon the basal forage source. We propose that this interaction arises from synergistic or antagonistic effects within the rumen environment. Specifically, the combination of SRU with different silages likely creates distinct nutritional milieus—particularly in terms of energy-nitrogen synchronization and fiber degradability—that differentially regulate rumen microbial population dynamics and biohydrogenation activity [64,65]. These shifts in the rumen metabolic landscape subsequently alter the flow of specific fatty acid isomers to the small intestine for absorption, thereby impacting the final profile deposited in muscle tissue. Future studies employing microbiome sequencing are warranted to elucidate the precise mechanisms driving this interaction.

Evaluating the relationship between the fatty acid composition of meat and human health is a focal point in meat quality research. While numerous evaluation indices exist, each possesses distinct merits and limitations. The NVI focuses primarily on the most abundant fatty acids (C16:0, C18:0, and C18:1n-9) but neglects PUFAs, which are crucial for health [23]. In contrast, the IA and IT account for major saturated and unsaturated fatty acids, distinguishing between atherogenic and thrombogenic saturated fatty acids and their anti-atherogenic, anti-thrombogenic unsaturated counterparts [24,57]. Consequently, IA and IT serve as indicators of platelet aggregation potential and coronary heart health, with lower values indicating greater benefits for human health. The HH assesses fatty acids related to cholesterol metabolism, specifically hypocholesterolemic fatty acids (C18:1n-9 and PUFA) versus hypercholesterolemic fatty acids (C12:0, C14:0, C16:0) [25]. The HPI, an inverse of IA, similarly reflects the impact of fatty acids on cardiovascular disease risk. Finally, the FLQ index utilizes EPA and DHA content to evaluate lipid quality. Notably, in this study, the significant interactions between silage type and nitrogen source on these indices (including IA, IT, HH, and HPI) imply that the efficacy of SRU in modulating health-related nutritional characteristics is contingent upon the basal silage type. Specifically, substituting SM with SRU in the SS-based diet showed no adverse effects on the health indices of lamb meat. In stark contrast, the same substitution strategy in the CS-based diet proved detrimental, resulting in a reduced health value of the meat products, although the precise mechanisms underlying this divergence remain unclear. Moreover, replacing CS with SS improved the NVI of lamb meat. This improvement was driven by significantly lower IT values in the SS group, irrespective of the nitrogen source (SM or SRU). Thus, it can be concluded that SS-fed lamb meat offers superior benefits for coronary and cardiovascular health. Additionally, the HH and FLQ results indicated a superior fatty acid profile in the SS group, which is more conducive to reducing blood cholesterol levels in consumers. Consequently, lambs fed sweet sorghum silage exhibited superior health-promoting value compared to those fed CS, and the application of SRU in CS diets specifically diminished the nutritional quality of the meat.

Additionally, several limitations of this study should be noted. First, the use of a single breed and sex restricts the extrapolation of these results to broader populations, as genetic and physiological differences can influence metabolic responses. Second, the study design included only one substitution ratio of SRU for SM and SS for CS, which prevents the evaluation of dose-dependent effects. Third, the relatively small sample size (n = 6 per treatment), although sufficient to detect significant differences in this trial, may limit the robustness of the conclusions. Future research should aim to validate these findings across different breeds and sexes, utilize a gradient of inclusion levels, and employ larger sample sizes to provide more comprehensive insights.

## 5. Conclusions

In conclusion, this study demonstrates that the substitution of corn silage with sweet sorghum silage significantly enhances the nutritional and functional quality of lamb meat. Fatty acid profile analysis showed that 9 categories were affected by the interaction between silage type and nitrogen source, while 10 and 0 categories were affected by silage type and nitrogen sources, respectively. This indicates that the impact of nitrogen supplementation is dependent on the forage base. Notably, sweet sorghum silage enhanced meat color, antioxidant capacity, and the fatty acid profile—particularly by increasing polyunsaturated fatty acids (LA, EPA, and DHA)—thereby offering greater health benefits despite the elevated n-6/n-3 ratio. In this context, SRU proved advantageous for enhancing meat tenderness compared to soybean meal; conversely, its application in corn silage diets compromised the health value of the meat, highlighting a specific limitation of SRU in this dietary regime. Overall, incorporating sweet sorghum silage and SRU into lamb diets offers a viable strategy to optimize meat quality, particularly in terms of tenderness, oxidative stability, and fatty acid composition.

## Figures and Tables

**Table 1:** Ingredients and composition of the TMR (dry matter basis, %).

Item	Treatments			
	CS		SS	
	SM	SRU	SM	SRU
TMR ingredients (%)				
Whole-corn silage	30.00	30.00	0.00	0.00
Sweet sorghum silage	0.00	0.00	30.00	30.00
Peanut vine	20.00	20.00	20.00	20.00
Corn	26.90	33.4	26.90	33.4
Soybean meal	15.85	10.35	15.85	10.35
Wheat bran	5.00	3.00	5.00	3.00
Sodium bicarbonate	0.25	0.25	0.25	0.25
Slow-release urea	0.00	1.00	0.00	1.00
Salt	0.40	0.40	0.40	0.40
Premix ^(1)^	1.60	1.60	1.60	1.60
Chemical composition of TMR				
Dry matter	91.85	92.03	92.11	92.29
Crude protein	13.56	13.75	13.31	13.50
Ether extract	2.98	2.96	2.93	2.90
Neutral detergent fiber	28.21	27.61	33.99	33.39
Acid detergent fiber	18.38	17.91	20.72	20.26
Metabolizable energy (MJ/kg) ^(2)^	10.34	10.28	9.97	9.91
Calcium	0.87	0.84	0.90	0.88
Phosphorus	0.42	0.40	0.40	0.39

Note: TMR, total mixed ration; CS, corn silage; SS, sweet sorghum silage; SRU, slow release urea. ^(1)^ The pre-mix provided the following nutrients per kg of the diet: 12,000 IU/kg Vitamin A, 3000 IU/kg Vitamin D3, 300 IU/kg Vitamin E, 8 mg/kg Copper, 75 mg/kg Iron, 32 mg/kg Zinc, 40 mg/kg Manganese, 0.58 mg/kg Iodine, 0.24 mg/kg Selenium, 0.16 mg/kg Cobalt. ^(2)^ Value of metabolizable energy was calculated according to the NRC.

**Table 2:** Results of LT muscle chemical composition response to silage type and nitrogen source.

Items	ST		NS		SEM	*p*-Value		
	CS	SS	SM	SRU		ST	NS	I×
Moisture	73.32	73.74	73.82	73.25	0.48	0.845	0.733	0.720
Crude protein	20.15	20.51	20.41	20.25	0.29	0.572	0.690	0.778
Ether extract	4.55 ^a^	3.88 ^b^	3.91	4.52	0.18	0.044	0.068	0.693
Ash	1.50	1.41	1.43	1.47	0.04	0.218	0.570	0.188

CS, corn silage; SS, sweet sorghum silage; SM, soybean meal; SRU, slow release urea; ST, silage type; NS, nitrogen source; I×, interaction effect between silage type and nitrogen source. ^a,b^ Lowercase superscripts indicate significant differences among silage types (*p* < 0.05).

**Table 3:** Effects of dietary silage type and nitrogen source on meat quality of Hu lamb.

Items	ST		NS		SEM	*p*-Value		
	CS	SS	SM	SRU		ST	NS	I×
pH_45min_	6.39	6.41	6.39	6.41	0.02	0.800	0.644	0.201
pH_24h_	6.07	6.00	6.02	6.04	0.04	0.322	0.786	0.103
L*	45.26	44.94	44.61	45.59	0.48	0.740	0.319	0.215
a*	14.24 ^b^	16.44 ^a^	15.51	15.17	0.42	0.011	0.350	0.916
b*	18.15	17.89	18.04	17.99	0.28	0.666	0.936	0.936
Drip loss (%)	2.30	2.17	2.22	2.45	0.04	0.177	0.735	0.907
Cooking loss (%)	33.11	34.36	33.30	34.16	0.47	0.206	0.380	0.963
WBSF (N)	63.04 ^a^	51.42 ^b^	63.73 ^A^	50.73 ^B^	2.76	0.015	0.007	0.326

L*, lightness; a*, redness; b*, yellowness; WBSF, Warner–Bratzler shear force. CS, corn silage; SS, sweet sorghum silage; SM, soybean meal; SRU, slow release urea; ST, silage type; NS, nitrogen source; I×, interaction effect between silage type and nitrogen source. ^a,b^ Lowercase superscripts indicate significant differences among silage types (*p* < 0.05). ^A,B^ Uppercase superscripts indicate significant differences among nitrogen sources (*p* < 0.05).

**Table 4:** Antioxidant enzymes activity affected by silage type and nitrogen source in LT muscle of *Hu* lamb (Unit: U/mg protein).

Items	CS		SS		SEM	*p*-Value		
	SM	SRU	SM	SRU		ST	NS	I×
GSH-Px	60.85 ^Nn^	90.57 ^Mm^	96.40 ^Mm^	89.55 ^Mm^	3.80	/	/	0.001
**Items**	**ST**		**NS**					
	**CS**	**SS**	**SM**	**SRU**				
CAT	4.94 ^b^	5.55 ^a^	5.25	5.24	0.15	0.054	0.963	0.394
T-AOC	5.46 ^b^	7.07 ^a^	6.15	6.38	0.26	0.002	0.613	0.330
SOD	40.56 ^b^	47.35 ^a^	43.99	43.92	1.37	0.013	0.977	0.573

T-AOC, total antioxidant capacity; SOD, superoxide dismutase; GSH-Px, glutathione peroxidase; CAT, catalase; CS, corn silage; SS, sweet sorghum silage; SM, soybean meal; SRU, slow release urea; ST, silage type; NS, nitrogen source; I×, interaction effect between silage type and nitrogen source. ^M,N^: Different uppercase letters indicate significant differences (*p* < 0.05) between different nitrogen sources within the same silage type. ^m,n^: Different lowercase letters indicate significant differences (*p* < 0.05) between different silage types under the same nitrogen source. ^a,b^ Means with different lowercase letters in the same row differ significantly (*p* < 0.05). “/” For parameters where a significant interaction was detected (*p* < 0.05), main effects was removed from the table.

**Table 5:** Fatty acid composition of lamb meat in response to dietary silage type and nitrogen source (unit: % of total FA).

Items	CS		SS		SEM	*p*-Value		
	SM	SRU	SM	SRU		ST	NS	I×
C16:0	30.46 ^Nm^	31.80 ^Mm^	28.73 ^Mn^	27.65 ^Mn^	0.384	/	/	0.009
C16:1 n-7 cis	1.33 ^Mm^	1.16 ^Mn^	1.51 ^Nm^	1.84 ^Mm^	0.073	/	/	0.030
C18:1 n-9 trans	1.33 ^Mm^	1.29 ^Mn^	1.29 ^Nm^	1.40 ^Mm^	0.019	/	/	0.039
C18:1 n-7 cis	0.85 ^Mm^	0.55 ^Mn^	0.79 ^Mm^	1.01 ^Mm^	0.061	/	/	0.023
C22:4 n-6 cis	1.65 ^Mn^	0.98 ^Mn^	3.02 ^Nm^	4.06 ^Mm^	0.276	/	/	0.003
SFA	54.12 ^Nm^	55.69 ^Mm^	53.24 ^Mm^	52.53 ^Mn^	0.298	/	/	0.005
PUFA	10.63 ^Mn^	9.23 ^Nn^	13.90 ^Mm^	14.59 ^Mm^	0.498	/	/	0.013
UFA	45.88 ^Mm^	44.30 ^Nn^	46.76 ^Mm^	47.47 ^Mm^	0.298	/	/	0.005
SFA/UFA	1.18 ^Nm^	1.26 ^Mm^	1.14 ^Mm^	1.11 ^Mn^	0.014	/	/	0.007
**Items**	**ST**		**NS**					
	**CS**	**SS**	**SM**	**SRU**				
C8:0	0.02	0.02	0.02	0.02	0.002	0.366	0.714	0.691
C10:0	0.01	0.01	0.01	0.01	0.001	0.271	0.605	0.167
C12:0	0.06	0.05	0.04	0.06	0.006	0.574	0.076	0.232
C13:0	ND	0.01	0.003	0.004	0.001		0.151	
C14:0	0.44 ^b^	0.94 ^a^	0.67	0.70	0.064	<0.001	0.700	0.588
C15:0	0.36	0.35	0.34	0.36	0.015	0.799	0.448	0.259
C17:0	0.76 ^b^	0.97 ^a^	0.84	0.89	0.038	0.004	0.443	0.456
C18:0	21.89	22.11	21.91	22.09	0.207	0.630	0.697	0.841
C20:0	0.17 ^a^	0.14 ^b^	0.15	0.16	0.006	0.001	0.331	0.558
C22:0	0.05	0.05	0.05	0.05	0.002	0.119	0.617	0.119
C23:0	ND	0.03	0.01	0.02	0.003		0.374	
C24:0	0.02	0.03	0.03	0.03	0.001	0.103	0.933	0.143
C14:1 n-5 cis	ND	0.05	0.02	0.04	0.008		0.135	
C15:1 n-5 trans	0.02	0.02	0.02	0.02	0.001	0.140	0.709	0.677
C16:1 n-7 trans	0.11	0.10	0.12	0.10	0008	0.651	0.446	0.671
C18:1 n-7 trans	0.69	0.70	0.67	0.72	0.026	0.972	0.417	0.085
C18:1 n-9 cis	30.88 ^a^	27.88 ^b^	29.48	29.27	0.345	<0.001	0.467	0.135
C20:1 n-9 cis	0.21	0.20	0.20	0.21	0.008	0.662	0.494	0.066
C18:3 n-3	0.42	0.45	0.42	0.45	0.011	0.181	0.297	0.194
C20:5 n-3 cis	0.19 ^b^	0.31 ^a^	0.26	0.24	0.017	<0.001	0.534	0.647
C22:5 n-3 cis	0.31	0.45	0.38	0.38	0.016	0.100	0.813	0.740
C22:6 n-3 cis	0.67 ^b^	0.83 ^a^	0.74	0.75	0.022	0.041	0.800	0.195
C18:2 n-6 cis	3.28 ^b^	4.36 ^a^	3.95	3.67	0.172	<0.001	0.319	0.121
C18:3 n-6 cis	0.26	0.20	0.24	0.22	0.010	0.092	0.366	0.578
C20:3 n-6 cis	0.31 ^b^	0.39 ^a^	0.35	0.35	0.011	<0.001	0.782	0.288
C20:4 n-6	2.51	3.04	2.91	2.64	0.148	0.068	0.354	0.281
C22:5 n-6 cis	0.67	0.68	0.68	0.67	0.024	0.792	0.909	0.955
MUFA	35.17 ^a^	32.87 ^b^	34.06	33.98	0.261	0.001	0.732	0.677
n-6/n-3	5.27 ^b^	6.03 ^a^	5.81	5.49	0.174	0.027	0.322	0.327

SFA, saturated fatty acids; MUFA, monounsaturated fatty acids; PUFA, polyunsaturated fatty acids; UFA, unsaturated fatty acids; ND, not detected. CS, corn silage; SS, sweet sorghum silage; SM, soybean meal; SRU, slow release urea; ST, silage type; NS, nitrogen source; I×, interaction effect between silage type and nitrogen source. ^M,N^: Different uppercase letters indicate significant differences (*p* < 0.05) between different nitrogen sources within the same silage type. ^m,n^: Different lowercase letters indicate significant differences (*p* < 0.05) between different silage types under the same nitrogen source. ^a,b^ Means with different lowercase letters in the same row differ significantly (*p* < 0.05). “/” For parameters where a significant interaction was detected (*p* < 0.05), main effects was removed from the table.

**Table 6:** Health-related nutritional indexes of lamb meat under different silage types and nitrogen sources.

Items	CS		SS		SEM	*p*-Value		
	SM	SRU	SM	SRU		ST	NS	I×
IA	0.70 ^Nm^	0.76 ^Mm^	0.69 ^Mm^	0.67 ^Mn^	0.01	/	/	0.009
IT	1.94 ^Nm^	2.07 ^Mm^	1.81 ^Mn^	1.75 ^Mn^	0.03	/	/	<0.001
HH	1.34 ^Mn^	1.25 ^Nn^	1.42 ^Mm^	1.47 ^Mm^	0.02	/	/	0.022
HPI	1.42 ^Mm^	1.32 ^Nn^	1.44 ^Mm^	1.50 ^Mm^	0.02	/	/	0.009
**Items**	**ST**		**NS**					
	**CS**	**SS**	**SM**	**SRU**				
NVI	1.70	1.78	1.74	1.74	0.02	0.056	0.931	0.210
FLQ	0.85 ^b^	1.14 ^a^	1.00	0.99	0.03	<0.001	0.830	0.539

NVI, nutrition value index; IA, index of atherogenicity; IT, index of thrombogenicity; HH, hypocholesterolemic/hypercholesterolemic ratio; HPI, health-promoting index; FLQ, fish lipid quality. CS, corn silage; SS, sweet sorghum silage; SM, soybean meal; SRU, slow release urea; ST, silage type; NS, nitrogen source; I×, interaction effect between silage type and nitrogen source. ^M,N^: Different uppercase letters indicate significant differences (*p* < 0.05) between different nitrogen sources within the same silage type. ^m,n^: Different lowercase letters indicate significant differences (*p* < 0.05) between different silage types under the same nitrogen source. ^a,b^ Means with different lowercase letters in the same row differ significantly (*p* < 0.05). “/” For parameters where a significant interaction was detected (*p* < 0.05), main effects was removed from the table.

## Data Availability

The original contributions presented in this study are included in the article. Further inquiries can be directed to the corresponding author.

## Abbreviations

The following abbreviations are used in this manuscript:

a*	redness
b*	yellowness
CAT	catalase
CS	corn silage
DM	dry matter
DHA	docosahexaenoic acid
EPA	eicosapentaenoic acid
FLQ	fish lipid quality
GSH-Px	glutathione peroxidase
HH	hypocholesterolemic/hypercholesterolemic ratio
HPI	health-promoting index
IA	index of atherogenicity
IT	index of thrombogenicity
L*	lightness
LT	*Longissimus Thoracis*
MUFA	monounsaturated fatty acids
NPN	non-protein nitrogen
NVI	nutrition value index
PUFA	polyunsaturated fatty acids
SFA	saturated fatty acids
SM	soybean meal
SOD	superoxide dismutase
SRU	slow-release urea
SS	sweet sorghum silage
T-AOC	total antioxidant capacity
TMR	total mixed ration
UFA	unsaturated fatty acids
WBSF	Warner–Bratzler shear force
